# Interprofessionelle Lehre in der Pharmazie: Beispiele zur Förderung der interprofessionellen Zusammenarbeit in der Ausbildung an der Philipps-Universität Marburg und am Klinikum Fulda

**DOI:** 10.1007/s00103-025-04042-4

**Published:** 2025-04-07

**Authors:** Jennifer Weber, Carsten Culmsee, Roland Radziwill

**Affiliations:** 1 Institut für Pharmakologie und Klinische Pharmazie, Philipps-Universität Marburg, Karl-von-Frisch-Straße 2, 35043 Marburg, Deutschland; 2 Apotheke und Ernährungszentrum, Klinikum Fulda gAG, Fulda, Deutschland

**Keywords:** OSCE, IPSTA, Medizin trifft Pharmazie, IPSTA-Pharm-Studie, Klinische Pharmazie, OSCE, IPSTA, Medicine meets pharmacy, IPSTA-Pharm-study, Clinical pharmacy

## Abstract

Eine immer komplexer werdende, patientenspezifische Gesundheitsversorgung benötigt neue Ansätze für die Qualifizierung zukünftiger Fachkräfte, wobei die Interprofessionalität und Kooperation der Heilberufe immer mehr in den Vordergrund rücken. Für eine gute Zusammenarbeit der verschiedenen Professionen im Arbeitsalltag ist es unerlässlich, diese Interprofessionalität bereits in der Ausbildung zu erlernen. Das Fach Klinische Pharmazie als „patientenorientierte Pharmazie“ hat die Ziele, die Arzneimittelanwendung an und durch Patientinnen und Patienten zu optimieren, die Arzneimitteltherapiesicherheit zu gewährleisten und so Gesundheit und Lebensqualität der Patientinnen und Patienten zu verbessern. Darüber hinaus wird durch eine Erweiterung der Lehrinhalte die Grundlage für die interprofessionelle Zusammenarbeit mit Ärztinnen und Ärzten bereits im Studium angelegt. Lehrformate, wie z. B. Übungen an Fall- und Rezeptbeispielen in Kleingruppen oder die *Objective Structured Clinical Examinations* (OSCEs), können den interprofessionellen Gedanken bereits im Studium vermitteln. An der Philipps-Universität Marburg lernen Medizin- und Pharmaziestudierende gemeinsam in interprofessionellen und fachbereichsübergreifenden Projekten wie „Medizin trifft Pharmazie“. Hier werden sowohl die medizinische Sicht auf Patientinnen und Patienten und ihre Erkrankungen als auch die pharmazeutische Sicht auf die medikamentöse Therapie und Arzneimittelrisiken mit vielfältigen Neben- und Wechselwirkungen betrachtet. Zusätzlich kann im Praktischen Jahr die Interprofessionalität durch die Teilnahme an interprofessionellen Ausbildungsstationen (IPSTAs) vertieft werden, wie ein Beispiel am Klinikum Fulda verdeutlicht.

## Abstract (en)

Increasingly complex and patient-specific healthcare requires new approaches to the qualification of future specialists, with interprofessionalism and cooperation between the healthcare professions becoming more and more important. In order for the various professions to work well together in everyday working life, it is essential to learn this interprofessionalism during training. The subject of clinical pharmacy as “patient-oriented pharmacy” aims to optimize patient medication and drug therapy safety and thus to improve the health and quality of life of patients. In addition, by expanding the teaching content, the basis for interprofessional collaboration with physicians is already laid during the training. Teaching formats, such as exercises on case reports and prescription examples in small student groups or objective structured clinical examinations (OSCEs), can convey the interprofessional concept during the courses. At the Philipps University of Marburg, students from the faculties of medicine and pharmacy study together in interprofessional and interdisciplinary projects such as “Medicine meets Pharmacy.” Here, both the medical perspective on patients and their illnesses and the pharmaceutical perspectives on drug therapy and drug risks with various side effects and interactions are considered. In addition, interprofessionalism can be further reinforced during the practical year by participating in interprofessional training wards (IPSTAs), as shown in an example at the clinic in Fulda.

## Hintergrund

Steigende Bevölkerungszahlen, chronische Krankheiten, ein zunehmender Fachkräftemangel, Lieferengpässe von Medikamenten sowie eine sich ständig weiterentwickelnde und immer komplexer werdende Gesundheitsversorgung benötigen neue Ansätze für die Qualifizierung der Fachkräfte. Apothekerinnen und Apotheker spielen in dieser komplexen Versorgungssituation eine zentrale Rolle im interprofessionellen Team und können mit ihrer Expertise zur individuellen Therapieoptimierung beitragen [1, 2]. Im interprofessionellen Team sind Apothekerinnen und Apotheker bereits in vielen Projekten erfolgreich integriert – sowohl im Krankhaus mit dem Antibiotic Stewardship (ABS), dem Closed-Loop-Medication-Management (CLMM) bzw. dem Apotheker auf Station als auch im ambulanten Bereich mit dem Qualifizierungskonzept Arzneimitteltherapiesicherheit in Apotheken (ATHINA), der praktischen Umsetzung des Zukunftskonzepts Arzneimittelinitiative in Sachsen und Thüringen ABDA-KBV-Modell/ARMIN oder den pharmazeutischen Dienstleistungen. Zusätzlich empfiehlt z. B. die Deutsche Interdisziplinäre Vereinigung für Intensiv- und Notfallmedizin (DIVI), dass Stationsapothekerinnen und -apotheker feste Mitglieder im interprofessionellen Team auf Intensivstationen sein sollten. Für eine gute Zusammenarbeit der verschiedenen Professionen im Arbeitsalltag ist es unerlässlich, Interprofessionalität bereits in der Ausbildung zu erlernen und anzuwenden. Problembasiertes Lernen, kompetenzorientierte Lehr- und Prüfungsformate sowie interprofessionelle Lehre und Kooperation der Heilberufe in Ausbildung und beruflicher Praxis rücken immer mehr in den Vordergrund [3–5]. In diesem Artikel werden Beispiele aus der Lehre der klinischen Pharmazie an der Philipps-Universität Marburg sowie am Klinikum Fulda aufgezeigt, in denen die Studierenden in Projekten wie „Medizin trifft Pharmazie“ und in strukturierten Lehr-Lernformaten sowie in interprofessionellen Ausbildungsstationen (IPSTAs) in der Klinik bereits im Studium bzw. im Praktischen Jahr (PJ) auf die zukünftige interprofessionelle Zusammenarbeit vorbereitet werden.

## Lehrformate der klinischen Pharmazie an der Philipps-Universität Marburg

Die klinische Pharmazie verfolgt als patientenorientierte Pharmazie die Zielsetzung, aufbauend auf pharmazeutisch-naturwissenschaftlichen Kenntnissen die Arzneimittelanwendung an und durch Patientinnen und Patienten zu optimieren und so deren Gesundheit und Lebensqualität zu verbessern. Die klinische Pharmazie kann so einen wichtigen Beitrag zur Arzneimitteltherapiesicherheit (AMTS) leisten und über die positiven Effekte für die Patientinnen und Patienten hinaus die interprofessionelle Zusammenarbeit mit den Ärztinnen und Ärzten verbessern sowie pharmakoökonomische Vorteile erzielen [6, 7]. Trotz der Implementierung des Fachs mit der letzten Änderung der Approbationsordnung für Apotheker im Jahr 2001 besteht für die klinische Pharmazie weiterhin erheblicher Entwicklungsbedarf. Die Umsetzung in Lehre und Forschung sowie in der Krankenversorgung mit den entsprechenden patientenorientierten Tätigkeiten in den Kliniken und in der öffentlichen Apotheke fallen im internationalen Vergleich deutlich zurück [8, 9]. Dabei eröffnet gerade das Fach Klinische Pharmazie bedeutende Chancen, sich von den tradierten Lehr- und Prüfungsformaten zu lösen und die in den Grundlagenfächern der Pharmazie vermittelten Kenntnisse und Fähigkeiten zu bündeln und in einer patientenorientierten Perspektive zu lehren und anzuwenden. An der Philipps-Universität Marburg reicht der Beginn der Ausbildung in klinischer Pharmazie bis ins Jahr 1994 zurück, als ein freiwilliges Seminar angeboten wurde. Seit 2007 ist das Fach mit einer eigenständigen Professur vertreten und hier besteht eine enge Zusammenarbeit in Forschung und Lehre mit interprofessionell eingebundenen Kooperationseinheiten der klinischen Pharmazie in den Kliniken in Kassel und Fulda.

## Übungen – Fallbeispiele und Rezeptbeispiele in Kleingruppen

Im Rahmen der Lehre der klinischen Pharmazie werden in Marburg Übungsformate in Kleingruppen (2–4 Studierende) angeboten, in denen Fallanalysen und Problemlösungen mithilfe pharmazeutischer Kompetenz im Mittelpunkt stehen. In diesen Übungen werden den Studierenden Fallbeispiele und Rezeptbeispiele mit kurzen Schilderungen von Erkrankungen, Medikation und aktuellen Problemen der Patientinnen und Patienten vorgelegt, die sie dann selbstständig mithilfe von Datenbanken, Medikationsanalysesoftware, Literatur und ihrem erworbenen Wissen gemeinsam lösen. In der Nachbesprechung in Gruppen von 20–25 Pharmaziestudierenden werden dann die Fälle kurz präsentiert und mit Unterstützung der Lehrenden diskutiert. Diese Formate der problembasierten Fallbearbeitungen werden von den Studierenden regelmäßig als überaus sinnvolle Lehreinheiten verstanden, in denen sie ihre pharmazeutischen Kenntnisse und Fähigkeiten in der gesamten Breite des Fachs selbstständig anwenden, verknüpfen und vertiefen. Hier werden auch ausdrücklich die Beratung der Patientinnen und Patienten sowie die interprofessionelle Kommunikation mit Ärztinnen und Ärzten und Pflegekräften mit in die Lösungsansätze eingearbeitet und in Rollenspielen vorgestellt.

## Praktische Übungen – Objective Structured Clinical Examination (OSCE)

Ein weiteres innovatives Lehr- und Prüfungsformat sind die *Objective Structured Clinical Examinations* (OSCE; [10, 11]). Im Studiengang Medizin sind OSCE-Formate schon sehr lange etabliert, um insbesondere die Kenntnisse und Fähigkeiten der Studierenden in einer aktiven Rolle und weit über Formate der schriftlichen oder mündlichen Wissensprüfung hinaus zu trainieren und zu bewerten. Die Studierenden werden hier in einem Prüfungsparcours in 10–12 Stationen anhand von kurzen Fallbeschreibungen und unter realistischen Zeitbedingungen problembasiert auf ihr pharmazeutisches und pharmakologisches Wissen geprüft. Zudem gibt es Stationen, in denen die direkte oder telefonische Kommunikation mit Ärztinnen und Ärzten, Pflegepersonal und Patientinnen und Patienten simuliert wird. Dafür stehen eigens für diese Formate ausgebildete und erfahrene Schauspielerinnen und Schauspieler aller Altersgruppen zur Verfügung, die Alltagssituationen in der Apotheke, im Krankenhaus und in der interprofessionellen Interaktion mit Ärztinnen und Ärzten und dem Pflegepersonal sehr realistisch simulieren können. Dies wird insbesondere dadurch gewährleistet, dass die OSCEs im Marburger interdisziplinären Skills Lab „Maris“, dem Zentrum für medizinische Lehre am Fachbereich Medizin, durchgeführt werden, in denen die Umgebungen in der Klinik, am Handverkaufstisch der Apotheke und telefonische sowie direkte Kontakte sehr realitätsnah nachgestellt werden können. Es stehen vollständig eingerichtete Krankenzimmer zur Verfügung, in denen die Simulationspatientinnen und -patienten in den Betten liegen und so vorgefunden werden, wie auf Station im Krankenhaus. Die Bewertung der Leistungen der Studierenden kann in speziellen Abhörräumen erfolgen, sodass die Bewertenden nicht von den Studierenden oder den Simulationspatientinnen und -patienten wahrgenommen werden. Die theoretischen und praktischen Stationen sind klar strukturiert und laufen nach einem festen Zeitplan ab, der von 2 Personen überwacht und mittels akustischer Signale gesteuert wird. Die Stationen mit den Simulationspatientinnen und -patienten bzw. interprofessioneller Kommunikation mit Ärztinnen und Ärzten und Pflegepersonal sind insgesamt für 6 min konzipiert. Nach einer kurzen Orientierung über die Aufgabe mithilfe eines kurzen Fallberichts haben die Studierenden 4 min Zeit für das Beratungsgespräch. Unmittelbar im Anschluss werten die Schauspielerinnen und Schauspieler und die Bewertenden nach einem vorbereiteten Punktesystem die Leistungen der Studierenden gemeinsam aus. Die Studierenden rotieren derweil zur nächsten Station, wo auf das Signal die Vorbereitungsphase und nach einem weiteren Signal das nächste Gespräch folgt. Den theoretischen Stationen stehen jeweils 6 min für die Bearbeitung der Aufgabe zur Verfügung, die unter Verwendung von Hilfsmitteln (Fachinformation, Rote Liste, Arzneimittel-Pocket-Formate) gelöst werden können. In diesem Setting können bis zu 60 Studierende pro Tag in den OSCEs ausgebildet und geprüft werden [10–12].

## Interprofessionelle Lehrformate im Studium – Medizin trifft Pharmazie

Die Forderung nach einer Verankerung der interprofessionellen Ausbildung und Zusammenarbeit in den Curricula der Gesundheitsberufe wird immer stärker [13, 14]. Die Weltgesundheitsorganisation (WHO) legte bereits 2010 Rahmenbedingung für die interprofessionelle Zusammenarbeit fest und verfolgt das Ziel, diese in Studium, Ausbildung und Praxis zu implementieren [15]. Das gemeinsame Lernen von Berufsgruppen, die im Berufsalltag zusammenarbeiten, soll durch gemeinsame Lehrformate und -veranstaltungen bereits in der Ausbildung umgesetzt werden, um die Voraussetzungen für die optimale Versorgung im Hinblick auf AMTS und Patientengesundheit anzulegen [16]. Obwohl eine interprofessionelle Zusammenarbeit eindeutig sinnvoll und notwendig erscheint, ist diese nicht fest in den Approbationsordnungen der Studiengänge festgeschrieben und somit nur an wenigen Universitäten implementiert. Im Medizinstudium erlernen die angehenden Ärztinnen und Ärzte Grundlagen der medikamentösen Versorgung der Patientinnen und Patienten; das Pharmaziestudium umfasst auch medizinische Grundlagen für zukünftige Apothekerinnen und Apotheker in den Fächern Krankheitslehre, Pharmakologie und Klinische Pharmazie – jeweils in geringem Umfang und überwiegend in theoretischem (Frontal‑)Unterricht. Beide Professionen sollten in der Berufsausübung die Sichtweise der anderen Berufsgruppe verstehen, ihre Kompetenz nutzen und zum Wohl der Patientinnen und Patienten eng zusammenarbeiten, was auch der Bundesverband der Pharmaziestudierenden in Deutschland e. V (BPhD) gemeinsam mit der Bundesvertretung der Medizinstudierenden in Deutschland e. V. (bvmd), dem Deutschen Berufsverband für Pflegeberufe und der Jungen Pflege fordern [17, 18]. Auch im „Masterplan Medizinstudium 2020“ wird eine Zusammenarbeit von Ärztinnen und Ärzten verschiedener Fachrichtungen, aber auch mit Angehörigen anderer Gesundheitsberufe im Hinblick auf die immer komplexer werdende Patientenbetreuung gefordert. Sinnvollerweise sollte hiermit schon während der Ausbildung begonnen werden [14, 19]. Die Robert-Bosch-Stiftung und das Institut für medizinische und pharmazeutische Prüfungsfragen (IMPP) setzen sich für eine Neuordnung der Aufgabenverteilung und Kompetenzzuweisungen der einzelnen Gesundheitsberufe ein und fordern eine interprofessionelle strukturierte Ausbildung [20]. Diskussionen über eine neue Approbationsordnung für Apothekerinnen und Apotheker laufen unter Federführung der Bundesapothekerkammer mit den betroffenen Verbänden. Hierbei wird neben einer fächerübergreifenden Lehre auch die Etablierung einer interprofessionellen Lehre angestrebt, damit das gegenseitige Verständnis und das Vertrauen in die Kompetenzen der eigenen und anderen Berufsgruppe bereits in der Ausbildung gestärkt werden können [21]. Im Positionspapier „Apotheke 2030“ wird ein interprofessionelles Netzwerk gefordert, in dem Apothekerinnen und Apotheker untereinander sowie mit anderen Gesundheitsberufen kollegial für eine bestmögliche Patientenversorgung zusammenarbeiten sollen [1]. Im Aktionsplan 2021–2024 wird eine Teamarbeit aller am Arzneimittelprozess beteiligten Berufsgruppen zur Sicherstellung der AMTS gefordert. Zur Umsetzung seien interprofessionelle Lehrveranstaltungen für das Studium zielführend, die etabliert werden müssen [22].

An der Philipps-Universität Marburg initiierte das Zentrum für medizinische Lehre im Jahr 2019 das interprofessionelle und fachbereichsübergreifende Projekt „Medizin trifft Pharmazie“. Hier lernen in 2 verschiedenen Unterrichtsformaten Medizinstudierende und Pharmaziestudierende gemeinsam: Sie erfahren exemplarisch in einer Visite am Patientenbett in der Klinik sowohl die medizinische Sicht auf Patientinnen und Patienten und ihre Erkrankungen als auch die pharmazeutische Sicht auf die medikamentöse Therapie und Arzneimittelrisiken mit vielfältigen Neben- und Wechselwirkungen. In einem Seminarformat erarbeiten Studierende beider Fachbereiche darüber hinaus in Kleingruppen Diagnosen und die mögliche Therapie anhand von komplexen, realistischen Fallbeispielen, die schriftlich vorliegen. Lehrende aus beiden Fachbereichen erörtern abschließend mit den Studierenden beider Studiengänge die in den Kleingruppen ausgearbeiteten Kasuistiken^1^.

## Interprofessionelle Ausbildungsstationen (IPSTA) im Krankenhaus

Über die oben skizzierten Lehr- und Lernformate im Rahmen des Studiums sind auch weiterführende Formate der fachübergreifenden Ausbildung im Rahmen des PJ, z. B. in IPSTAs, als Ausbildungseinheiten innerhalb einer Normalstation möglich [23]. Ein interprofessionelles Team aus Studierenden und Auszubildenden übernimmt hierbei eigenständig die Patientenbetreuung und das Stationsmanagement unter Supervision von erfahrenen Lernbegleiterinnen und Lernbegleitern der beteiligten Berufsgruppen [24]. In das interprofessionelle Team können alle Studierenden und Auszubildenden integriert werden, die an der Patientenversorgung beteiligt sind. IPSTAs gibt es in Deutschland bereits seit dem Jahr 2017 und die Anzahl steigt jährlich an, allerdings werden Pharmazeutinnen und Pharmazeuten im Praktikum (PhiPs) im interprofessionellen Team noch nicht regelhaft berücksichtigt. Selbst im Jahr 2024 waren PhiPs nur vereinzelt und oft nur stundenweise im Team integriert [24, 25]. Die häufigsten Professionen im Team bilden deutschlandweit Medizinstudierende im PJ und Pflegeauszubildende [26–30]. Auszubildende der Physio- und Ergotherapie finden sich ebenfalls nur an wenigen Standorten im Team wieder [31–34].

Am Klinikum Fulda wurden im Januar 2022 gleichzeitig 3 IPSTAs eröffnet. Diese befinden sind in der Nephrologie, der Allgemein- und Viszeralchirurgie (AVC) und der Kinderklinik. Das interprofessionelle Team am Klinikum Fulda besteht aus Medizinstudierenden im PJ (PJler), Pflegeauszubildenden – möglichst im dritten Ausbildungsjahr – und PhiPs. Die PJler und Pflegeauszubildende arbeiten im Früh- und Spätdienst, wohingegen die PhiPs den Tagdienst abdecken und sowohl mit dem Früh- als auch dem Spätdienst zusammenarbeiten. Eine IPSTA-Einheit dauert i. d. R. 4 Wochen und beginnt mit einem Einführungstag. Hierbei lernt sich das interprofessionelle Team untereinander sowie die Station und deren Aufgaben kennen. Es werden Kommunikationsregeln sowie eine strukturierte Übergabe besprochen und das Team erhält eine Übergabe der Patientinnen und Patienten, die es ab dem Folgetag eigenständig betreut. Das Team betreut 2 oder ggf. 3 Zimmer mit bis zu 6 Patientinnen und Patienten unter Supervision von erfahrenen Lernbegleiterinnen und Lernbegleitern. Diese sind unterstützend und beobachtend tätig und agieren im Stationsalltag u. a. motivierend und kommunikationsfördernd. Sie gestalten die Lehr- und Lernumgebung mit und dienen allgemein als Vorbild [35]. Sie greifen erst dann ein, wenn das IPSTA-Team Hilfe einfordert oder es behandlungstechnisch notwendig wird, jedoch soll das Team selbst Behandlungsoptionen und Therapien erarbeiten [35]. Jede der 3 IPSTA-Stationen hat ein zusätzliches IPSTA-Zimmer geschaffen, indem sich das Team während der 4 Wochen aufhält. Unumgänglich sind hierbei genügend PC-Arbeitsplätze, eigene Telefone, ein eigener Verbandswagen und Geräte zur Diagnostik. Jedes Team kann sich das Zimmer individuell nach seinen Bedürfnissen einrichten und gestalten. Durch eigene Telefone und genügend Arbeitsplätze kann der interprofessionelle Gedanke auch räumlich umgesetzt werden.

Da die PhiPs 6 Monate in der Krankenhausapotheke arbeiten und etwa 8 IPSTA-Einheiten pro Klinik und Jahr stattfinden, können sie an jeder IPSTA innerhalb des PJ teilnehmen. Die IPSTA-Zeiträume werden von einem Koordinationsteam festgelegt und an die Ausbildungsabläufe der Berufsgruppen, so gut es geht, angepasst. Zur Vorbereitung der PhiPs auf eine IPSTA bleiben in der Apotheke durch die vordefinierten Zeitpunkte meist nur 2 Wochen, was dem PJ-Beginn Mai und November geschuldet ist. Die PhiPs werden sowohl in die Aufgaben einer modernen Krankenhausapotheke als auch in die Aufgaben auf einer IPSTA eingearbeitet. Sie erfahren eine strukturierte Einarbeitung anhand eines Einarbeitungsplanes und zusätzlich Fachgespräche über definierte Themen, die sich in Allgemein‑, Basis- und Vertiefungswissen gliedern. Bis zum ersten IPSTA-Tag müssen die Themen aus dem allgemeinen Teil und die Themen des Basiswissens durch Fachgespräche mit Apothekerinnen und Apothekern vermittelt werden. Die Kommunikation mit Patientinnen und Patienten, der Umgang mit Laborparametern oder der richtige Umgang mit der Arzneimittelinformation sind nur einige Beispiele, die im allgemeinen Teil sowohl theoretisch als auch praktisch am Patientenbeispiel vermittelt werden. Das Basis- und Vertiefungswissen zielt auf die adäquate Arzneimitteltherapie von definierten Krankheitsbildern ab. Zum Basiswissen zählen Krankheitsbilder, die den PhiPs im Stationsalltag oft begegnen, wie z. B. Diabetes mellitus, Antikoagulation, Schmerztherapie und kardiale Erkrankungen wie Hypertonie, Herzinsuffizienz oder koronare Herzerkrankung. Die stationsbezogenen Fachgespräche zu den Krankheitsbildern enthalten u. a. die leitliniengerechte Therapie, inklusive der eingesetzten Medikamente und deren klinischer Relevanz, sowie ggf. ein therapeutisches Drug-Monitoring und das perioperative Management. Zusätzlich lernen die PhiPs bezogen auf die Aufgaben der Krankenhausapotheke das Arzneimittelsortiment kennen, werden in relevante IT-Programme und in die Arzneimittelanamnese sowie das elektive Patientenmanagement (EPM) eingearbeitet.

Der Tagesablauf der PhiPs auf einer IPSTA unterteilt sich in individuelle berufsspezifische und interprofessionelle Abschnitte. Je nach Fachbereich unterscheiden sich die Tagesabläufe geringfügig. Auf der chirurgischen IPSTA (Tab. 1) startet der Tag mit einer interprofessionellen Übergabe und Visite. Änderungen bezüglich der Medikation oder Therapie werden anschließend im Team besprochen und die Aufgaben untereinander verteilt. Die PhiPs übernehmen vormittags die pharmazeutische Aufnahme in Form der Arzneimittelanamnese von sowohl Patientinnen und Patienten im EPM als auch von notfallmäßig auf die IPSTA aufgenommenen Patientinnen und Patienten. Da Patientinnen und Patienten je nach freier Bettenkapazität auf die IPSTA-Zimmer verteilt werden, ist es wichtig, auch elektive Patientinnen und Patienten durch die PhiPs aufnehmen zu lassen, um alle Aufnahmegespräche selbstständig durchgeführt zu haben. Die Medikation wird hierbei vollständig mit der Patientin oder dem Patienten besprochen und mögliche Unklarheiten geklärt, bevor der/die PhiP diese in die elektronische Patientenakte (ePa) einträgt und auf die Hausmedikation umstellt. Die Eingabe und Umstellung werden anschließend durch eine Apothekerin oder einen Apotheker kontrolliert. Eine interprofessionelle Entlassung erfolgt bis 11 Uhr, wobei jeweils ein Bundeseinheitlicher Medikationsplan (BMP) gemeinsam von den PJlern und PhiPs erstellt und den Patientinnen und Patienten mitgegeben wird. Stationäre Änderungen oder neu angesetzte Medikamente werden bei dem pharmazeutischen Entlassgespräch hervorgehoben und offene Fragen geklärt. Nachmittags erfolgt eine interprofessionelle Übergabe an den Spätdienst, inklusive eines Vortrags eines IPSTA-Teilnehmenden zu einem ausgewählten Thema. Anschließend findet eine zweite Visite des Spätdienstes statt, bei der die PhiPs ebenfalls anwesend sind.

**Tab. 1:** Tagesablauf der Pharmazeutinnen und Pharmazeuten im Praktikum auf einer chirurgischen interprofessionellen Ausbildungsstation (*IPSTA)*

Tagesablauf der Pharmazeutinnen und Pharmazeuten im Praktikum auf einer chirurgischen interprofessionellen Ausbildungsstation (IPSTA)	
7:00 Uhr	Start des IPSTA-Tages
7:00–7:45 Uhr	Gemeinsame Visite mit interprofessioneller Patientenbesprechung
7:45–12:00 Uhr	Pharmazeutische Aufnahmegespräche elektiver Patientinnen und Patienten der Allgemein- und Viszeralchirurgie (AVC) im elektiven Patientenmanagement (EPM); Eingabe und Umstellung der Medikation auf die Hausmedikation
7:45–ca. 15:00 Uhr	Pharmazeutische Aufnahmegespräche mit neuen IPSTA-Patientinnen und -Patienten
Bis 11:00 Uhr	Entlassungen der Patientinnen und Patienten; gemeinsame Erstellung eines Bundeseinheitlichen Medikationsplanes (BMP) und führen von pharmazeutischen Entlassgesprächen
13:30–14:15 Uhr	Interprofessionelle Übergabe an den Spätdienst, Kurzvortrag eines IPSTA-Teilnehmenden zu einem ausgewählten Thema (montags bis donnerstags), gemeinsamer Wochenrückblick (freitags)
14:15–14:30 Uhr	Gemeinsame Visite

Der Tagesablauf auf der nephrologischen Station unterscheidet sich lediglich in der Aufnahme elektiver Patientinnen und Patienten, da es diese auf der nephrologischen Station nur vereinzelt gibt (Tab. 2).

**Tab. 2:** Tagesablauf der Pharmazeutinnen und Pharmazeuten im Praktikum auf einer nephrologischen interprofessionellen Ausbildungsstation (*IPSTA)*

Tagesablauf der Pharmazeutinnen und Pharmazeuten im Praktikum auf einer nephrologischen interprofessionellen Ausbildungsstation (IPSTA)	
8:00 Uhr	Start des IPSTA-Tages
8:30–9:30 Uhr	Gemeinsame Visite mit interprofessioneller Patientenbesprechung
9:30–10:00 Uhr	Nachbesprechung der Patientinnen und Patienten und ggf. gemeinsame Medikationsanpassung
8:00–16:00 Uhr	Pharmazeutische Aufnahmegespräche mit neu aufgenommenen IPSTA-Patientinnen und -Patienten
Bis 13:00 Uhr	Entlassungen der Patientinnen und Patienten; gemeinsame Erstellung eines Bundeseinheitlichen Medikationsplanes (BMP) und Führen von pharmazeutischen Entlassgesprächen
11:00–13:00 Uhr	Bearbeitung von Arzneimittelanamnesen
13:30–14:00 Uhr	Interprofessionelle Übergabe an den Spätdienst
14:00–14:15 Uhr	Präsentation eines IPSTA-Teilnehmenden zu einem ausgewählten Thema (montags bis donnerstags), gemeinsamer Wochenrückblick (freitags)

Auf der pädiatrischen Station ist der Tagesablauf kontinuierlich angepasst worden, da für viele PhiPs die Aufgabenfelder und Tagesabläufe ohne eine (komplexe) Medikation schwierig umsetzbar waren. In den ersten beiden IPSTA-Wochen ist der Tagesablauf mit dem der nephrologischen Station vergleichbar. Hier sollen die PhiPs den Ablauf einer pädiatrischen Station (Tab. 3), die Kommunikation mit Kindern und deren Eltern, pädiatrische Krankheitsbilder und Besonderheiten in der Medikation bei Kindern kennenlernen. Ab der dritten Woche sind die PhiPs telefonisch erreichbar und nehmen an einer wöchentlichen Visite sowie an gemeinsamen Fallbesprechungen teil. Eine telefonische Erreichbarkeit über die gesamte Zeit garantiert eine interprofessionelle Zusammenarbeit, sodass bei pharmazeutischen Fragestellungen oder Aufnahmegesprächen von Kindern mit Medikamenten die PhiPs auch in den Wochen 3 und 4 in das Team integriert sind. In diesen beiden Wochen sollen die PhiPs die die Kinderklinik betreffenden Aufgaben in der Apotheke kennenlernen. Hierzu gehört die Plausibilisierung der Antibiotikaanforderungen, die Plausibilisierung und Zubereitung von parenteraler Ernährung im Sterillabor, die galenische Plausibilisierung und Herstellung von Pulverbriefchen, Kapseln oder Suspensionen für Kinder sowie die Arzneimittelinformation in der Pädiatrie.

**Tab. 3:** Tagesablauf der Pharmazeutinnen und Pharmazeuten im Praktikum auf einer pädiatrischen interprofessionellen Ausbildungsstation (*IPSTA)*

Tagesablauf der Pharmazeutinnen und Pharmazeuten im Praktikum auf einer pädiatrischen interprofessionellen Ausbildungsstation (IPSTA)	
8:00 Uhr	Start des IPSTA-Tages
8:30–9:30 Uhr	Gemeinsame Visite mit interprofessioneller Patientenbesprechung
08:00–16:00 Uhr	Pharmazeutische Aufnahmegespräche mit neuen IPSTA-Patientinnen und -Patienten
13:00 Uhr	Fallbesprechungen jeden Dienstag und Donnerstag
13:30–14:00 Uhr	Interprofessionelle Übergabe an den Spätdienst: Kurzvortrag eines IPSTA-Teilnehmenden zu einem ausgewählten Thema (montags bis donnerstags), gemeinsamer Wochenrückblick (freitags)
Ab der *3.* IPSTA-Woche:	
–	1 × wöchentlich und nach Bedarf: pharmazeutische Visite
	Teilnahme an den Fallbesprechungen jeden Dienstag und Donnerstag um 13:00 Uhr
	Telefonische Erreichbarkeit der Pharmazeutinnen bzw. der Pharmazeuten im Praktikum von 8–16 Uhr

Das IPSTA-Team nutzt die interprofessionelle Zeit, um Aufgaben der eigenen und anderen Berufsgruppe kennenzulernen und Aufgaben gemeinsam zu erledigen. So stellen die PhiPs an ausgewählten Tagen gemeinsam mit den Pflegeauszubildenden die Medikamente für die Patientinnen und Patienten und können Inhalte der Arzneiformlehre vermitteln und erklären. Die Pflegeauszubildenden und PhiPs unterstützen die PJler bei der Diagnostik und den Untersuchungen.

Aus den Tagesabläufen der PhiPs ergeben sich deren Aufgabengebiete (Tab. 4). Diese gliedern sich in die Aufnahme neuer Patientinnen und Patienten durch pharmazeutische Aufnahmegespräche, die sowohl eine Arzneimittelanamnese sowie eine Medikationsanalyse beinhalten, die Aufgaben während des stationären Aufenthalts sowie die pharmazeutischen Entlassgespräche mit der Erstellung des BMP, wobei das Augenmerk auf geänderte oder neue Medikamente sowie Einnahmezeitpunkte oder mögliche Wechselwirkungen gelegt wird.

**Tab. 4:** Aufgabengebiete der Pharmazeutinnen und Pharmazeuten im Praktikum auf einer interprofessionellen Ausbildungsstation (IPSTA)

Aufgabengebiete der Pharmazeutinnen und Pharmazeuten im Praktikum auf einer interprofessionellen Ausbildungsstation (IPSTA)
*Patientenaufnahme*
Pharmazeutische Aufnahmegespräche, inkl. Arzneimittelanamnese von IPSTA-Patientinnen und -Patienten
Eingabe der Medikation in die elektronische Patientenakte (ePA) und Umstellung auf die Hausmedikation
Medikationsanalyse, inkl. Interaktionschecks
Überprüfung der Medikation auf Plausibilität
Anpassung der Medikation an die Nierenfunktion
*Stationärer Aufenthalt*
Teilnahme an Visiten und interprofessionellen Übergaben
Änderungen der Medikation im Team besprechen und Dokumentation in die Verlaufsdokumentation
Ggf. Medikation auf Sondengängigkeit prüfen
Tägliche Laborwertkontrolle und Medikationsanalyse
Antibiotikamanagement (Dosierung, Indikation, Dauer, Mikrobiologie)
Thromboseprophylaxe und therapeutische Antikoagulation
Ernährungszustand beachten und ggf. Therapie initiieren
Therapieoptimierung und Beratung zu Arzneimittelfragen
*Entlassung*
Erstellen von Entlassempfehlungen im interprofessionellen Team (Medizinstudierende im Praktischen Jahr und Pharmazeutinnen und Pharmazeuten im Praktikum)
Erstellen des Bundeseinheitlichen Medikationsplanes (BMP) mit Ergänzung der Indikation und Hervorhebung neu angeordneter bzw. stationär geänderter Medikamente
Im Gespräch mit Patientinnen und Patienten wird der BMP besprochen und besondere Hinweise auf Einnahmezeitpunkte, Interaktionen und Wechselwirkungen mit anderen Medikamenten oder Nahrungsmitteln gegeben
Rückversicherung, ob Fragen offen sind
Evtl. Mitgabe von Zusatzinformationen: Blutdrucktagebuch, Patienteninformationen zu neuen Arzneimitteln oder Zytostatika

Durch die sehr unterschiedlichen Fachrichtungen sind auf jeder IPSTA andere Schwerpunkte und damit auch Lernziele vorhanden. Der zusätzliche Lerngewinn für die PhiPs auf jeder IPSTA ist individuell an die jeweilige Station angepasst und soll innerhalb der 4 Wochen von den PhiPs eigenständig erarbeitet werden. Durch wöchentliche Besprechungen sowohl auf Station als auch mit den Lernbegleiterinnen und Lernbegleitern in der Apotheke werden der Stand der Lernziele verfolgt und offene Fragen geklärt. Allgemeine Ziele sind beispielsweise das Erlernen einer guten Teamkommunikation oder das Verstehen der Verantwortlichkeiten der eigenen und der anderen Berufsgruppe. Stationsspezifische Ziele werden stetig angepasst und umfassen beispielsweise das sichere Anpassen von Medikamenten an die Nierenfunktion, das Verstehen des perioperativen Managements oder das Anwenden der Arzneimittelinformation im pädiatrischen Bereich.

Am Ende jeder IPSTA stellen die PhiPs den komplexesten Patientenfall den anderen PhiPs und Mitarbeiterinnen und Mitarbeitern der Apotheke vor. So können sie ihr neu erlangtes Wissen umsetzten und durch Aufarbeitung des Falls und spätere Rückfragen die Lernziele festigen.

Um einen reibungslosen Ablauf der IPSTA zu gewährleisten, sind die Lernbegleiterinnen und Lernbegleiter (Apothekerin/Apotheker) in der ersten IPSTA-Woche bei jeder Visite, jeder Besprechung und Nachbesprechung sowie bei Patientengesprächen dabei. Je nach Bedarf ist die aktive Präsenz auch in Woche 2 und 3 vorhanden. Sie sind anschließend immer telefonisch erreichbar, sodass die PhiPs auch nach der intensiven Betreuung oder bei akuten Fragen Rücksprache halten können. Nach der Eingabe der Medikation, größeren Therapieentscheidungen oder vor Vorträgen muss grundsätzlich Rücksprache gehalten werden.

Um die Auswirkung einer IPSTA sowohl auf PhiPs als auch auf Patientinnen und Patienten zu untersuchen, wird die IPSTA mit PhiPs wissenschaftlich im Rahmen einer Promotion zum Thema „Einbindung von Pharmazeutinnen und Pharmazeuten im Praktikum auf fachlich unterschiedlichen interprofessionellen Ausbildungsstationen“ begleitet. Diese wird vom Institut für Pharmakologie und Klinische Pharmazie der Philipps-Universität Marburg betreut. Das Hauptziel der Promotion ist die Erarbeitung eines Curriculums für PhiPs mit dem Schwerpunkt der interprofessionellen Zusammenarbeit. Zusätzliche Nebenziele sind die Messung des Kompetenz- und Wissenszuwachses der PhiPs auf einer IPSTA und die Untersuchung des Wissens der Patientinnen und Patienten in Bezug auf ihre Medikation nach interprofessioneller Zusammenarbeit und pharmazeutischen Entlassgesprächen. Da es deutschlandweit bzw. im deutschsprachigen Raum bislang keine wissenschaftlichen Daten zu der Einbindung von PhiPs auf einer IPSTA gibt, soll diese Arbeit erste Wirksamkeitsbelege darlegen. Eine Kontrollgruppe von PhiPs anderer Kliniken ohne IPSTA wird zum Vergleich gegenübergestellt. Beide Gruppen füllen zu definierten Zeitpunkten selbstentwickelte Tests für Wissen und interprofessionelle Kompetenz aus, die im Nachgang nach einem definierten Bewertungsbogen ausgewertet werden. Ebenfalls dienen Patientinnen und Patienten der Normalstation ohne interprofessionelle Behandlung und pharmazeutische Entlassgespräche als Kontrollgruppen.

Eine dauerhafte Integration der PhiPs in das interprofessionelle Team konnte am Klinikum Fulda bereits erreicht werden. Im Rahmen der wissenschaftlichen Begleitung konnten hierbei bereits erste Hinweise auf einen Wissens- und Kompetenzzuwachs gegeben werden. Die vollständige Auswertung und Veröffentlichung folgt mit Beendigung der Promotion.

Durch regelmäßige Feedbackgespräche sowohl von den IPSTA-Teilnehmenden im wöchentlichen Rückblick als auch im Jour fixe mit allen Lernbegleiterinnen und Lernbegleitern alle 2 Wochen werden die Zusammenarbeit und Organisation stetig optimiert.

## Fazit

Für eine höhere AMTS und eine bessere Patientenversorgung ist eine interprofessionelle Zusammenarbeit unumgänglich. Das Fach Klinische Pharmazie bietet bereits im Studium die Möglichkeit, den Grundstein für eine interprofessionelle Zusammenarbeit zu setzen. Durch Erweiterung der Lehrinhalte mit Übungen anhand von Fallbeispielen und Rezepten oder der Etablierung von OSCEs kann die Interprofessionalität früh vermittelt werden. Anschließend ist im PJ eine Integration der PhiPs in das interprofessionelle Team einer IPSTA sinnvoll. Die PhiPs erlernen zusätzliche Ausbildungsinhalte und können ihr pharmazeutisches Wissen vertiefen und erweitern. Sie lernen die eigenen Kompetenzen, aber auch die der anderen Berufsgruppe kennen und können ihre Rolle im Team verstehen. Das Beispiel Fulda zeigt, dass die Integration von PhiPs auf einer IPSTA im Rahmen eines strukturierten Curriculums möglich und sinnvoll ist. Gleichzeitig steht die Forderung des bvmd und des BPhD nach einer interprofessionellen Ausbildung im Raum. Die bundesweite Umsetzung im Studium und im Praktikum sollte im Rahmen der Approbationsordnung festgeschrieben werden.

## Footnotes

1 https://www.youtube.com/watch?v=hpWvfSeb2uA; letzter Zugriff: 24.02.2025.
